# Recurrent gene duplication in the angiosperm tribe Delphinieae (Ranunculaceae) inferred from intracellular gene transfer events and heteroplasmic mutations in the plastid *matK* gene

**DOI:** 10.1038/s41598-020-59547-6

**Published:** 2020-02-17

**Authors:** Seongjun Park, Boram An, SeonJoo Park

**Affiliations:** 1 Institute of Natural Science, Yeungnam University, Gyeongsan, Gyeongbuk 38541 South Korea; 2 Department of Life Sciences, Yeungnam University, Gyeongsan, Gyeongbuk 38541 South Korea

**Keywords:** Plant evolution, Plant sciences, Chloroplasts

## Abstract

The study of intracellular gene transfer may allow for the detection of interesting evolutionary processes such as ancient polyploidization. We compared 24 plastid genomes (plastomes) from tribe Delphinieae, one from tribe Nigelleae and one from tribe Ranunculeae, including five newly sequenced genomes. The functional transfers of the plastids *rpl32* and *rps16* to the nucleus in tribe Delphinieae were identified. Unexpectedly, we discovered multiple divergent copies of the nuclear-encoded plastid *rpl32* in the genus *Aconitum*. Phylogenetic and synonymous substitution rate analyses revealed that the nuclear-encoded plastid *rpl32* underwent two major duplication events. These ancient gene duplication events probably occurred via multiple polyploidization events in *Aconitum* between 11.9 and 24.7 Mya. Furthermore, our sequence rate analysis indicated that the eight plastid-encoded *rpl* subunits in *Aconitum* had a significantly accelerated evolutionary rate compared to those in other genera, suggesting that highly divergent paralogs targeted to the plastid may contribute to an elevated rate of evolution in plastid *rpl* genes. In addition, heteroplasmy of the plastid *matK* from two *Aconitum* species suggested the existence of potentially functional plastid maturases in its plastome. Our results provide insight into the evolutionary history of the tribe Delphinieae.

## Introduction

Gene duplication (GD) has played an important role in eukaryotic evolution by generating evolutionary novelty^1^. GD occurs through various mechanisms, including whole-genome duplication (WGD), tandem duplication, transposon-mediated duplication, segmental duplication, and retroduplication^2^. After a duplication event, the duplicated genes are usually functionally redundant, resulting in pseudogenization and gene loss^3^. However, if the presence of duplicated genes is beneficial, the copies may acquire new functions via subfunctionalization^4^ and neofunctionalization^1^. Furthermore, duplicated copies can be retained without acquiring novel functions due to gene dosage effects^5^. In plants, WGD or polyploidization provides a notable opportunities for adaptive radiation, speciation and diversification^6–9^. Many plant lineages, such as Brassicaceae, Fabaceae, Poaceae, and Solanaceae, have undergone one or more genome-doubling events, leading to increased species richness^10^. Detecting and evaluating WGDs in plants is challenging because of rapid gene losses or diploidization over time^11,12^. Moreover, recurrent WGDs during plant evolution can promote chromosomal rearrangement^13,14^, which complicates the identification of events.

The tribe Delphinieae Schröd. is one of the largest tribes within Ranunculaceae; it comprises >650 species, accounting for ~26% of all Ranunculaceae species^15^. The species in this tribe exhibit anatomical and morphological diversity in their flowers^16,17^. Polyploidization has played a critical role in the speciation and diversification of tribe Delphinieae^18^. This tribe exhibits an enormous range of polybasic chromosome numbers (x = 6, 7, 8, 9, 10, and 13; main basic number x = 8), and ploidy levels from diploid (2n = 12) to octoploid (2n = 64)^19^. Moreover, many Delphinieae species, especially those of the genus *Aconitum*, form species complexes by producing interspecific hybrids in their natural habitats^20^. Thus, this tribe offers an intriguing system to study the dynamics of WGD, including GD, but the understanding of WGD within Delphinieae remains limited. To date, 25 complete Delphinieae plastomes have been sequenced, mainly from the genus *Aconitum* (23), one from the genus *Consolida*, and one from the genus *Gymnaconitum*. The sequenced Delphinieae plastomes generally range in size from 155.5 to 157.4 kb with a quadripartite organization. Losses of translation initiation factor A (*infA*) and two plastid-encoded ribosomal protein genes (*rpl32* and *rps16*) have been documented in the 25 completely sequenced Delphinieae plastomes^21–23^. In angiosperms, intracellular gene transfer (IGT) to the nucleus is an ongoing evolutionary process^24^ and is associated with the pseudogenization or loss of plastid coding genes. The IGT of *infA* and *rpl32* from the plastid to the nucleus has been documented multiple times across angiosperms^25–27^. However, in the case of *rps16*, the nuclear-encoded plastid *rps16* was supplanted by a nuclear-encoded mitochondrial *rps16* that had previously been transferred from the mitochondria to the nucleus via IGT^28^. In addition to gene losses, a truncated plastid-encoded *matK* lacking its C-terminal portion has been documented in the *A. austrokoreense* and *A. chiisanense* plastomes^29^. The truncation of protein-coding genes can lead to impairment or loss of gene function, but the function of this gene has not been evaluated.

The study of IGT events can help determine the evolutionary history of GD within tribe Delphinieae. In this study, we generated complete plastome sequences for five species (*Aconitum pseudolaeve*, *Consolida orientalis*, *Delphinium maackianum*, *Staphisagria macrosperma*, and *Nigella damascena*) to improve plastome sampling in tribe Delphinieae. Combining these data with previously published plastomes, we examined the phylogenetic distribution of gene losses and identified functional transfers to the nucleus via IGT or gene substitution. These surveys revealed multiple copies of the nuclear-encoded *rpl32* among *Aconitum* species, suggesting at least one GD event in the genus. We analyzed the distributions of synonymous substitution rates (*d*_S_) among the paralogs of 10 *Aconitum* species to estimate the number of GD events. The evolutionary fates of the duplicated copies and the potential effects on nucleotide substitution were also evaluated. In addition, we evaluated the functional properties of the truncated plastid-encoded *matK* gene.

## Results

### General features of new plastome sequences

The complete plastomes for four species (*A. pseudolaeve*, *C. orientalis*, *D. maackianum*, and *S. macrosperma*) from tribe Delphinieae and *N. damascena* from tribe Nigelleae were determined by paired-end Illumina reads with deep coverages, ranging from 372x to 1,004x (Supplementary Table S1 and Supplementary Fig. S1). Each of the five plastomes was assembled into a circular molecule consisting of a large single copy (LSC) and a small single copy (SSC) region separated by a pair of inverted repeats (IRs), and the plastome sizes ranged from 154,484 bp to 155,915 bp. In tribe Delphinieae, the four plastomes shared a set of genes that encoded 77 proteins, 30 tRNAs, and 4 rRNAs. The ribosomal protein L32 (*rpl32*) and the ribosomal protein S16 (*rps16*) appeared to have been lost or pseudogenized in the plastomes of all four species (Supplementary Fig. S1A). In the case of *N*. *damascena*, *rpl32* was also lost, but the *rps16* gene was an intact gene in its plastome. A contraction at the IR_B_/SSC boundary resulted in a translocation of *ycf1* to the SSC region, and an IR_A_/SSC expansion included the C-terminal portion of *ndhF* (59 bp), generating a truncated *ndhF* fragment in IR_B_ (Supplementary Fig. S1B).

### Potential functional transfer of two plastid genes to the nucleus in tribe delphinieae

**Figure 1:**
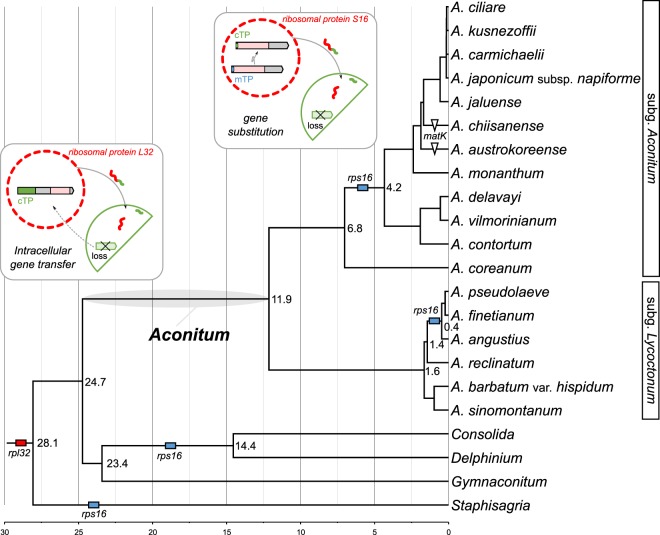
Phylogenetic distribution of gene content among tribe Delphinieae with divergence times. Numbers at nodes indicate divergence time estimates in Mya. Schematic diagram of functional transfers of the *rpl32* and *rps16* genes from plastids to the nucleus by intracellular gene transfer and gene substitution, respectively. Pink boxes indicate the conserved domains of each gene. The green and blue boxes in the N-terminus indicate the chloroplast (cTP) and mitochondrial (mTP) transit peptides, respectively. The figures were constructed in BEAST2 v2.5.1 (https://www.beast2.org/) and InkScape v0.92.2 (https://inkscape.org).

To understand the evolutionary history of gene losses in tribe Delphinieae, we reconstructed phylogenetic relationships and estimated divergence times. The phylogenetic tree, which was inferred from 77 plastid genes with 67,650 aligned nucleotide positions, provided strong support for relationships within tribe Delphinieae (Supplementary Fig. S2). Our analysis suggests that the split between *Aconitum* and the other genera occurred at 24.7 Mya (95% highest posterior density (HPD) = 14.49–34.88; Supplementary Fig. S3). Within the genus *Aconitum*, the subgenera *Aconitum* and *Lycoctonum* diverged at 11.9 (95% HPD = 6.35–18.45). Comparative analyses of the tribe Delphinieae plastomes revealed that the loss of the *rpl32* gene was shared by tribe Delphinieae, but the loss of the *rps16* gene was lineage-specific (Fig. 1). The phylogenetic distribution indicated that the truncation of plastid-encoded *matK* had occurred independently in *A. chiisanense* and *A. austrokoreense* (Fig. 1). We found that all the sampled Delphinieae plastomes contained the plastid-encoded *infA* gene, which was previously annotated as a gene loss in some *Aconitum* species (Supplementary Fig. S4).

The transcriptome data of four Delphinieae species (*Aconitum carmichaelii, C. orientalis*, *D. maackianum*, and *S. macrosperma*) provide strong evidence for potential functional transfers of the *rps16* and *rpl32* genes (Supplementary Table S2). The plastid-encoded *rps16* has been functionally replaced by the nuclear-encoded mitochondrial *rps16*, whereas the plastid-encoded *rpl32* has been subject to functional transfer to the nucleus (Fig. 1). In tribe Delphinieae, a gene substitution for the plastid *rps16* was also identified because the *rps16*-like transcripts were not detected by a “blastn” search against the plastid-encoded *rps16* gene of *R. macranthus*; however, two different transcripts were detected using the query sequence of the nuclear-encoded plastid *rps16* (*M. truncatula*, AB365526). Phylogenetic analyses of the *rps16* sequences from four Delphinieae species and the nuclear-encoded plastid and mitochondrial *rps16* (AB365527) of *M. truncatula* indicated two different origins for the transcripts (Supplementary Fig. S5).

Interestingly, the *A. carmichaelii* transcriptome contains two divergent *rpl32* copies with 88.9% nucleotide sequence identity (Supplementary Fig. S6A), whereas *Consolida*, *Delphinium*, and *Staphisagria* each have only one transcript for *rpl32*. All the predicted open reading frames (ORFs) have a transcript peptide with a conserved ribosomal L32 domain (Supplementary Table S2). To confirm the multiple versions and evaluate their intron-containing or intronless status, a nuclear copy was amplified from the *A. pseudolaeve* by specifically targeting the nuclear-encoded *rpl32*. RT-PCR analysis revealed that *A. pseudolaeve* also contains at least two different versions of *rpl32* with no premature stop codons or frameshift-producing insertions or deletions (indels) and that both copies are transcribed (Supplementary Fig. S6B). Alignment of the DNA and RNA nucleotide sequences revealed that the nuclear-encoded *rpl32* is an intronless gene (Supplementary Fig. S6B).

### *Aconitum* contains multiple versions of the nuclear-encoded plastid *rpl32* gene

**Figure 2:**
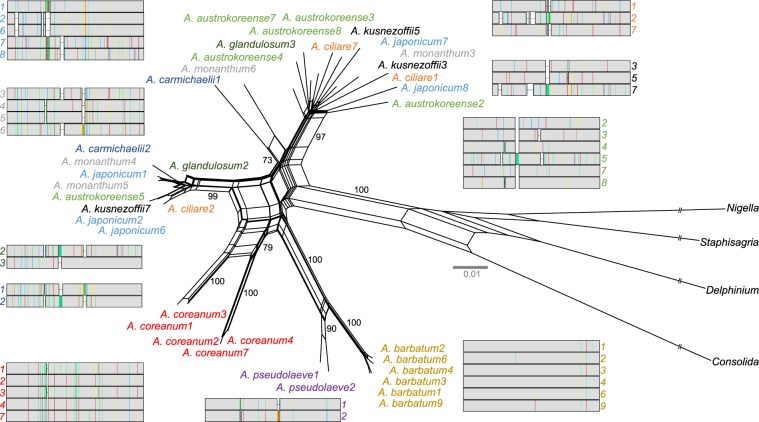
Phylogenetic network of nuclear-encoded plastid *rpl32* homologs. The numbers after each species indicate the paralogs of *rpl32*. The colored taxon name corresponds to the colored numbers after the alignment. The colored bars on the alignment map indicate nucleotide sites that differ from the consensus sequence (red, A; blue, C; yellow, G; green, T) (See Supplementary Fig. S6 for more details). The figures were constructed in SplitsTree v4.10 (https://en.bio-soft.net/tree/SplitsTree.html) and Geneious R7 v7.1.8 (https://www.geneious.com), and modified in InkScape v0.92.2 (https://inkscape.org).

Eight additional species of *Aconitum* representing two subgenera were surveyed for identification of the nuclear-encoded plastid *rpl32* copy using PCR, cloning, and Sanger sequencing. We found that multiple copies were also present as intact genes or pseudogenes in most of the examined *Aconitum* species (Table 1 and Supplementary Fig. S6C–J). Notably, all 10 examined *Aconitum* species contained at least two copies of the intact nuclear-encoded plastid *rpl32*, which ranged from 408 bp to 441 bp in length (Table 1). The nucleotide sequence alignments of the nuclear copies showed that they were highly divergent (88.5% nucleotide sequence identity), containing single nucleotide polymorphisms (SNPs) and indels (Table 1 and Fig. 2). No gene conversion between the paralogs of the nuclear-encoded *rpl32* gene was detected by GENECONV.

**Table 1:** Nuclear-encoded *rpl32* homologs in the genus *Aconitum*. I, intact gene; P, pseudogene. The number in parentheses indicates the copy number of pseudogene.

	Intact copy number	Length	Align.	Identity	GC content	Type	Gene integrity
*A. austrokoreense*	6	429–438	450	86.2–97.9	52.2–53.8	Indels/SNPs	I & P (1)
*A. barbatum*	6	444	444	99.1–99.8	54.5–55.2	SNPs	I
*A. carmichaelii*	2	435, 438	450	88.9	53.2, 53.8	Indels	I
*A. ciliare*	3	408–429	441	83.0–95.3	51.7–52.2	Indels/SNPs	I & P (3)
*A. coreanum*	5	435–441	441	93–99.8	50.1–52.2	Indels/SNPs	I
*A. glandulosum*	2	435, 438	450	87.8	53.6, 53.7	Indels	I & P (3)
*A. japonicum*	5	408–441	441	81.9–99.8	51.7–53.1	Indels/SNPs	I & P (2)
*A. kusnezoffii*	3	408–429	441	82.5–96.3	51.6–52.9	Indels/SNPs	I & P (3)
*A. monanthum*	4	429–438	450	88.4–99.8	52.4–53.4	Indels/SNPs	I & P (1)
*A. pseudolaeve*	2	435, 438	441	95.5	54.6, 55.6	Indels	I

**Figure 3:**
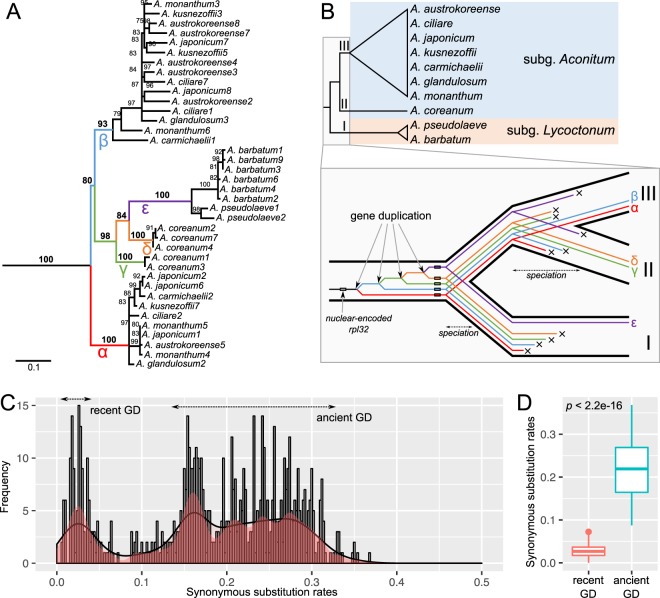
Nuclear-encoded plastid *rpl32* gene duplication events. (**A**) ML gene tree based on the nuclear-encoded *rpl32* nucleotide sequences. The numbers after each species indicate the paralogs of *rpl32*. Bootstrap support values > 70% are shown on the branches. (**B**) Phylogenetic relationships within *Aconitum*. Ancestral gene duplications are first followed by a speciation and then by asymmetric gene losses. Three lineages have one or two ancestral paralogs. (**C**) Histograms of the *d*_S_ distribution of the nuclear-encoded *rpl32* homologs derived from recent and ancient GD events. The *d*_S_ distribution was fitted using Gaussian mixture models. The *d*_S_ peaks corresponding to each GD. (**D**) Comparison of sequence divergence between homologs from two different GD events. The figures were constructed in IQ-TREE v1.6.2 (https://iqtree.org), R v3.4.2 (https://r-project.org) and InkScape v0.92.2 (https://inkscape.org).

A Neighbor-Net analysis was used to show the clustering of the *Aconitum rpl32* sequences (Fig. 2). The paralogous copies from *A. coreanum*, *A. barbatum*, and *A. pseudolaeve* were grouped together, respectively, but the others were scattered within two clusters. Phylogenetic analyses of the nuclear-encoded plastid *rpl32* homologs from tribe Delphinieae revealed five main lineages with high bootstrap support (Fig. 3A and Supplementary Fig. S7). We hereafter refer to them as alpha (α), beta (β), gamma (γ), delta (δ), and epsilon (ε). The phylogenetic relationships of the nuclear *rpl32* sequences were not consistent with the plastid phylogenomic relationships (Fig. 3B and Supplementary Fig. S2). For example, the *rpl32* gene tree showed that clade III (corresponding to lineage α) was basal to the other clades. The next diverging clade was lineage β (clade III), followed by lineage γ (clade II). The subgenus *Lycoctonum* (clade I: lineage ε), which is an early-diverging lineage in *Aconitum*, was nested within clade II (lineage δ). To examine rate variation in the duplicated genes, the nonsynonymous (*d*_N_) and synonymous (*d*_S_) substitution rates for their homologs were computed using the *rpl32* gene tree as a constraint tree. We discovered higher synonymous divergence in the homologs of lineage ε than in those of the other lineages (Supplementary Fig. S8). The synonymous branch for lineage ε was 3.0–8.6 times longer than those for the other lineages. Three branches with *d*_N_/*d*_S_ > 1 were detected in lineage β (*A. kusnezoffii3*&5 and *A. japonicum7*, Supplementary Fig. S8), but likelihood ratio tests (LRTs) showed that none of them were significantly different (*p* = 1.00 after Bonferroni correction) (Supplementary Table S3). Taken together, the phylogenetic incongruence and the different substitution rates of the homologs indicated that each lineage had an ancestral *rpl32* copy unique to that lineage and that the ancestral *rpl32* copies were shared with a single ancestral lineage.

Thus, we propose a model for the evolution of the nuclear-encoded *rpl32* gene after IGT to account for the gene duplication (GD) events in the *Aconitum* lineage (Fig. 3B and Supplementary Fig. S7). In this model of the ancestral GD, the most parsimonious interpretation is that the nuclear *rpl32* gene first underwent multiple GD events (at least four times) and that five *rpl32* paralogs existed in the common ancestor of *Aconitum* until speciation event that split the ancestor into two new lineages (Fig. 3B and Supplementary Fig. S7). Then, one of the new lineages split into two additional new lineages, followed by stochastic loss. After the ancestral GD, the gene tree showed recurrent lineage-specific duplication and speciation events, and the patterns of the recent GD events were more complicated (Supplementary Fig. S7). The histograms of the synonymous substitution rates for the homologous pairs supported two potential major GD events (Fig. 3C). We observed one and two peaks in the recent and ancestral GDs, respectively. Four peaks in the ancestral GD were detected by the adjusted Gaussian (Fig. 3C), supporting the model. The duplicated copies derived from the ancestral GD event exhibited greater divergence than those from the recent GD event (Wilcoxon rank-sum test, *p* < 0.01) (Fig. 3D). The divergence time estimates suggested that the ancestral GD events occurred between 11.9 and 24.7 Mya and that the recent GD events occurred ≤ 4.2 Mya (Fig. 1).

**Figure 4:**
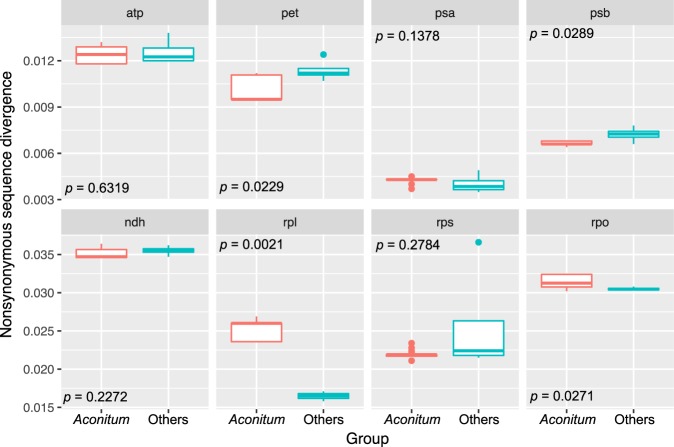
Box plots of *d*_N_ values for plastid functional gene groups in *Aconitum* and other genera. The box represents values between quartiles, the solid lines extend to the minimum and maximum values, and the horizontal lines in the boxes show the median values. *Aconitum*, red; other genera, blue.

Furthermore, we estimated the *d*_N_ and *d*_S_ substitution rates for the plastid-encoded gene and compared them to address whether the nucleotide substitution rates for the plastid-encoded ribosomal protein large (*rpl*) subunits in *Aconitum* were elevated by the presence of multiple versions of the nuclear-encoded *rpl32*. Only the *d*_N_ values for the plastid *rpl* subunits that assemble the nuclear-encoded *rpl32* protein were significantly higher in *Aconitum* than in other genera (Wilcoxon rank-sum test, *p* < 0.01; Fig. 4 and Supplementary Fig. S8).

### Heteroplasmic variation in the plastid-encoded maturase K

**Figure 5:**
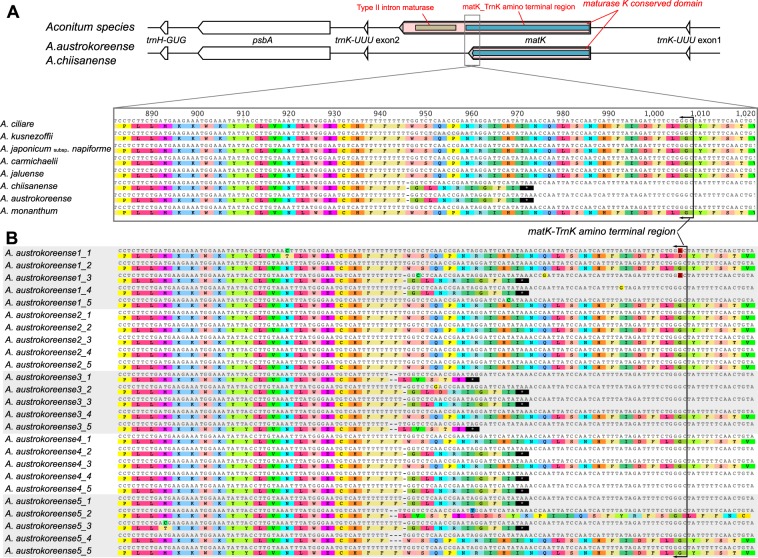
Characterization of the plastid-encoded *matK* genes of *Aconitum*. (**A**) Schematic diagram of the genetic region surrounding *matK* in *A. austrokoreense* and *A. chiisanense* compared with other *Aconitum* species. Boxes inside the *matK* gene indicate the conserved domains (matK_TrnK amino terminal region: blue, Type II intron maturase: brown). Nucleotide sequence alignment of the *matK* hotspot region in eight *Aconitum* species. (**B**) Nucleotide sequences of the *matK* copies from five *A. austrokoreense* individuals. The solid lines (black) indicate the end of the conserved domain of the matK_TrnK amino terminal region.

The two published *Aconitum* plastomes, those of *A. austrokoreense* and *A. chiisanense*, harbored a truncated plastid maturase K (*matK*) gene in their genomes. Comparisons of the *matK* copies from these species with those of related species revealed that former shared a single-base (T) deletion (position 946) that generated a premature stop codon (Fig. 5A). Investigation of the conserved domain revealed that the *matK* copies had approximately half of the gene, which contained only the conserved “*matK_TrnK amino terminal region*” domain and lacked the “*Type II intron maturase”* domain (Fig. 5A). To confirm this variation, we examined the polymorphisms in the plastid-encoded *matK* by cloning and Sanger sequencing an additional five individuals of *A. austrokoreense* (Fig. 5B). High polymorphic variation was detected in the homopolymeric (T) region of the 25 isolates of the five *A. austrokoreense* individuals; this region ranged in length from 8 to 11 bp (Fig. 5B). Two nontriplet deletions caused frameshifts, but a triplet “TTT” deletion resulted in a single amino acid loss (phenylalanine).

## Discussion

We have shown that all analyzed *Aconitum* plus *Consolida*, *Delphinium*, *Gymnaconitum*, and *Staphisagria* species lack the plastid-encoded *rpl32* gene in their plastomes, suggesting an ancient loss in the ancestor of tribe Delphinieae. In contrast, the phylogenetic distribution of the sampled plastomes indicated that the plastid-encoded *rps16* gene has been lost several times within the tribe. The transcriptomes indicated that the two plastid genes, *rpl32* and *rps16*, were lost after their functional replacement by IGT and gene substitution in the nucleus, respectively. These evolutionary processes suggest that the inactivation of two plastid genes is favored after nuclear gene activation. It is well known that polyploidization and hybridization contribute to *Aconitum* species diversity^18–20^. Our findings raise fundamental questions about the fates of transferred genes in a polyploid plant. If IGT events occurred before polyploid formation, gene doubling would occur via polyploidization. If multiple organelle-targeted copies are present, what are the potential effects of these multiple nuclear copies on the nucleotide substitution rates of the subunits encoded in the plastids?

Surprisingly, we discovered that all the sampled *Aconitum* species contained multiple versions of the nuclear-encoded plastid *rpl32*. We observed that the *rpl32* paralogs from each *Aconitum* species were highly divergent, having both SNPs and indels. However, the *rpl32* phylogeny showed sporadic distribution and phylogenetic incongruence (Fig. 3A). These phenomena generally indicate that independent events took place in each lineage or that stochastic losses with vertical transfer happened after GD events. An alternative explanation for this phenomenon is horizontal gene transfer within the genus. The clustering of *rpl32* homologs and the *d*_S_ distribution signals clearly demonstrated that the multiple copies originated from duplications (ancient GD), which may be associated with WGD. The presence of *rpl32* pseudogenes in several *Aconitum* species suggested that their sporadic distribution is due to inactivation and eventual loss over time. Given the WGD signal and chromosome number, the genus *Aconitum* may have undergone multiple events of polyploidization followed by diploidization in the ancient lineage. Many diploid *Aconitum* (2n = 16) species contain two or more *rpl32* paralogs, supporting the hypothesis of *Aconitum* paleopolyploidy. In contrast, it is unclear whether the recent GD is the result of WGD or smaller scale segmental or single-gene duplication. The recurrent GD and losses make it difficult to determine the mechanisms. The nuclear-encoded *rps16* could also have been affected by a recent genome doubling event, but we did not find evidence for GD of *rps16*. This finding supports the hypothesis that recent GD events may involve multiple evolutionary factors rather than only WGD. However, it is possible that the duplicated *rps16* genes were lost ancestrally, given their redundancy. Transcriptome data from other species are required to obtain a full understanding of the evolutionary history of WGD among the genus *Aconitum*. Duplicated plastid-targeted genes are reduced to a single-copy gene^30^, and reciprocal gene loss occurs between duplicated genes, which lead to divergence^31^. The nuclear *rpl32* sequences found in some *Aconitum* species represent pseudogenes (Supplementary Fig. S6). Thus, the nuclear-encoded plastid *rpl32* paralogs are expected to be retained as single-copy genes over time.

Highly divergent mutations in the nuclear-encoded genes that target organelles can drive compensatory changes in associated organelle genes^32–34^. In plants, cytonuclear coevolution has been observed in plastid protein complexes that include subunits encoded in the nuclear and plastid genomes^35,36^. After protein synthesis of the nuclear-encoded plastid *rpl32* in the cytosol, they must be imported into the plastids and assembled into plastid-localized multisubunit complexes (i.e., with the products of *rpl2*, *rpl14*, *rpl16*, *rpl20*, *rpl22*, *rpl23*, *rpl33*, and *rpl36*). We tested the effects of the nuclear *rpl32* copies on substitution rate changes in the plastid-encoded *rpl* subunits. The plastid-encoded *rpl* subunits of the genus *Aconitum* had highly accelerated nonsynonymous substitution rates relative to the corresponding rates of the other analyzed genera (Fig. 4). These results lead us to conclude that highly divergent multiple copies of the nuclear-encoded *rpl32* may trigger compensatory changes in the eight plastid-encoded *rpl* subunits. However, alternative mechanisms, such as relaxation of functional constraints^37^ and cytonuclear incompatibilities^36,38^, can contribute to accelerated sequence evolution in the *Aconitum* plastid-encoded *rpl* subunits. Further studies that include organelle ribosomes as well as nuclear-encoded regulatory factors are needed.

Maturase K (*matK*) is encoded within the intron of tRNA^Lys^ (*trnK-UUU*) in the plastids^39^. The *matK* protein is essential for the splicing of group II introns^40^, which target seven *cis*-spliced introns from *atpF*, *rpl2*, *rps12*, tRNA^Val^ (*trnV-UAC*), tRNA^Ile^ (*trnI-GAU*), tRNA^Ala^ (*trnA-UGC*), and *trnK-UUU*^41^. Truncated *matK* genes have been documented in two *Aconitum* species^29^ that both lack the C-terminal portion. Our analyses revealed that the *matK* genes of these two species lacked the conserved *Type II intron maturase* domain at the C-terminal (Fig. 5), suggesting a loss of functionality. To maintain its function in the plastids, *matK* would need to be functionally replaced by the nucleus. However, previous studies have suggested the parallel loss of all introns and plastid *matK*^42–44^. The two *Aconitum* plastomes retain the seven target introns, indicating the necessity for *matK* in their plastids. Interestingly, PCR cloning and Sanger sequencing revealed heteroplasmic variation in the *matK* sequences of five *A. austrokoreense* individuals (Fig. 5). The presence of a triplet nucleotide deletion or no mutation at the same position indicated that they probably encode functional proteins in the *A. austrokoreense* plastid. These sequences that suggested the functionality of *matK* are from the plastome and indicate that *A. austrokoreense* contains at least two versions in its plastome. High coverage sequencing of the *A. austrokoreense* plastome^29^ suggests that the truncated *matK* copies exist predominantly in its plastome. However, we cannot exclude the possibility that some sequences were amplified from a nuclear plastid DNA (NUPT) or a mitochondrial plastid DNA (MIPT). Complete mitochondrial and nuclear genomes (including transcriptome) sequences are needed to better address this phenomenon. Heteroplasmic mutations are identified either within a cell or among cells; they can occur due to replication slippage, recombination, cytonuclear interaction, or diverse environmental factors^45–48^. The frameshift mutation in the *matK* genes from the two *Aconitum* plastomes may be associated with chlorophyll deficiency^49^.

## Conclusions

This study provides valuable insights into the patterns of genome evolution across tribe Delphinieae. In particular, duplication of the nuclear-encoded *rpl32* after ITG was found in all the sampled *Aconitum* species of tribe Delphinieae. Our phylogenetic analysis showed five diverse clades of *rpl32* homologs, implying that at least five paralogs existed in the common ancestor of the genus *Aconitum*. The *d*_S_ distribution signal suggested that additional *rpl32* paralogs have arisen via duplication in recent *Aconitum* evolution. The ancient GD appears more likely to have been caused by WGD than by a single event, whereas the recent GDs have involved multiple processes, such as WGD, single-gene duplication, hybridization, or horizontal gene transfer. Rate analyses supported the hypothesis that selection for compensatory changes in response to multiple divergent *rpl32* paralogs contributes to an elevated rate of evolution in plastid *rpl* subunits. In addition, heteroplasmy in the *matK* within and among individuals is identified, but the evolutionary forces driving this phenomenon is nuclear. To obtain a comprehensive understanding of the plastid heteroplasmy among *Aconitum* genomes, multiple genome sequences from a population are required.

## Methods and Methods

### Genome sequencing, assembly and annotation

Total genomic DNA (gDNA) of *Aconitum pseudolaeve*, *Consolida orientalis*, *Delphinium maackianum*, *Staphisagria macrosperma*, and *Nigella damascena* was isolated from fresh leaf tissues using the DNeasy Plant Mini Kit (Qiagen, Hilden, Germany) following the manufacturer’s protocol (Supplementary Table S4). The five gDNAs were sequenced using the Illumina HiSeq 2500 sequencing platform (Illumina, San Diego, CA), generating approximately 6 Gb of paired-end (PE) reads from a 550-bp insert library.

The PE reads were assembled *de novo* with Velvet v1.2.10^50^ using multiple *k*-mers (69 to 91) on a 32-core 3.33 GHz Linux work station with 512 GB of memory. Five plastomes were determined following the methods in Park *et al*.^51^. We annotated the plastomes using a BLAST-like algorithm in Geneious R7 v7.1.8 (www.geneious.com)^52^ with the genes of the model plant tobacco (*Nicotiana tabacum*, NC_001879) as the reference and confirmed the open reading frames (ORFs). All tRNA genes were predicted using tRNAscan-SE v2.0.3^53^ and ARAGORN v1.2.38^54^. The plastomes were deposited in GenBank (accession number MN648400-MN648404). Circular plastome maps were drawn with OGDRAW v1.3.1 (chlorobox.mpimp-golm.mpg.de/OGDraw.html)^55^.

### Phylogeny and divergence times

A single alignment data set was concatenated with 77 protein-coding gene alignments from 22 species of tribe Delphinieae and two outgroups, *N. damascena* and *Ranunculus macranthus* (Supplementary Table S5). The individual gene alignments were generated based on the back-translation approach with MAFFT v7.017^56^ in Geneious R7. The maximum likelihood (ML) tree was inferred from the concatenated data set under each of 17 partitioning schemes (Supplementary Table S6) using IQ-TREE v1.6.2^57^ with the ultrafast bootstrap algorithm^58^ (1,000 replicates). Divergence times were inferred in BEAST2 v2.5.1^59^ using a concatenated alignment data set that excluded the two outgroups. A calibration point estimated for tribe Delphinieae^60^ (mean = 28.95, SD = 5, range 20.7–37.2 Ma) was used with a normal prior distribution as the root age constraint.

### Identification of functional transfer to the nucleus

Total RNA was isolated from fresh leaves of *C. orientalis*, *D. maackianum*, and *S. macrosperma* using the methods of Ghawana *et al*.^61^ and treated with DNase I (Invitrogen, USA). The RNAs were sequenced using the Illumina HiSeq 2500 sequencing platform, generating approximately 6 Gb of PE reads. To evaluate potential IGT sources, transcriptomes from *C. orientalis*, *D. maackianum*, and *S. macrosperma* were assembled *de novo* with Trinity v2.5.1^62^. In addition, *Aconitum carmichaelii* (SRR6225422) and *Nigella sativa* (SRR341997) were included. Nuclear-encoded protein genes (*rpl32* and *rps16*) for plastids were identified by “blastn” searches (*e*-value cutoff of 1e-10) using BLAST + v2.7.1^63^, employing plastid-encoded *rpl32* and *rps16* from *R. macranthus* as the query sequences. We also used the *Medicago truncatula* (AB365526) nuclear-encoded plastid *rps16*, which was supplanted by the nuclear-encoded mitochondrial *rps16*^28^. The chloroplast transit peptide (cTP) and its cleavage site were predicted by TargetP v1.1^64^. The NCBI Conserved Domain Database (CDD) was used for functional domain annotation^65^.

### Survey of variability in the nuclear-encoded plastid *rpl32*

To better understand the evolutionary fate of the nuclear-encoded plastid *rpl32*, eight *Aconitum* species were sampled (Supplementary Table S7). Total genomic DNA was isolated from herbarium specimens using the methods of Allen *et al*.^66^. To detect the nuclear *rpl32* copies, polymerase chain reaction (PCR) was carried out using the total genomic DNA. Primer pairs were designed based on sequences of the nuclear *rpl32* copies from *A. carmichaelii* using Primer3^67^ in Geneious R7 (forward: CCATGGCSACATCTCTACTACCMA and reverse: CACCACAAAGTAGATGGGGCTTGG). The PCR was 50 μl in volume, including 38.75 μl of distilled water, 5 μl of 10 × Taq Reaction Buffer, 1 μl of 10 mM dNTPs, 0.25 μl of DiaStar Taq polymerase (5 units/μl, Solgent Co., Daejeon, South Korea), 1 μl of each primer (10 pmol/μl), and 1 μl of total genomic DNA (20 ng). All reactions included an initial denaturation step (95 °C for 2 min), 35 cycles of denaturation (95 °C for 20 s), annealing (60 °C for 40 s), extension (72 °C for 1 min) and final extension (72 °C for 5 min). The PCR products were purified using a PCR purification kit (MGmed, South Korea) following the manufacturer’s protocol. First, we sequenced a product from *A. pseudolaeve* using an ABI 3730xl DNA Analyzer (Applied Biosystems, California, USA) at Solgent Co. The results showed a pattern of mixed nonspecific PCR products. Thus, the purified products from all exampled *Aconitum* species were cloned using the T-Blunt PCR Cloning Kit (Solgent Co., Daejeon, South Korea) following the manufacturer’s protocol. White colonies were picked, and direct colony PCRs were performed using Solg *h*-Taq DNA polymerase (Solgent Co., Daejeon, South Korea) with the M13 (−20) forward/reverse primers. The nucleotide sequences of isolated clones were determined by an automated DNA sequencer. The data set was aligned at the protein level by MAFFT using the translation-align function in Geneious R7. Neighbor-Net splits graphs of the nuclear-encoded *rpl32* data were constructed using SplitsTree v4.10^68^ with uncorrected *p* distance^69^. The gene tree was inferred from the nuclear-encoded *rpl32* paralog data set using IQ-TREE with the ultrafast bootstrap algorithm (1,000 replicates). Gene duplication events were inferred using Zmasek and Eddy’s algorithm^70^ in MEGA X^71^.

To confirm that the two nuclear *rpl32* copies were transcribed, total RNA isolation for *A. pseudolaeve* was performed as described above. Reverse transcription (RT)-PCR was performed using ImProm-II Reverse Transcriptase (Promega, USA) with random hexamers. PCR amplification was carried out with primer pairs specific to the nuclear-encoded *rpl32*. PCR purification and sequencing were performed as described above.

### Estimation of sequence divergence

Nonsynonymous and synonymous substitution rates were calculated in PAML v4.8^72^ using the ML tree as a constraint tree. Analyses were performed using the CODEML program, employing the F3 x 4 codon frequency model, and gapped regions were excluded with the “cleandata = 1” option. The estimations of rate variation were performed on the concatenated gene sets for the functional groups ATP synthase (*atp*), cytochrome b6f (*pet*), NADH-plastoquinone oxidoreductase (*ndh*), photosystem I (*psa*), photosystem II (*psb*), RNA polymerase (*rpo*), and ribosomal protein small (*rps*) and large (*rpl*) subunits. Likelihood ratio tests (LRTs) were performed to test the *d*_N_/*d*_S_ changes. The null model was fixed across the entire tree, whereas an alternative model allowed different values of *d*_N_/*d*_S_ for branches in the phylogenetic tree.

To test for evidence of selection acting on duplicated genes, the *d*_N_ and *d*_S_ values for the nuclear-encoded *rpl32* were calculated. We also estimated the *d*_S_ values between the pairs of *rpl32* homologs, and Gaussian mixture models were fitted using R v3.4.2^73^ to identify significant peaks. Statistical analyses were conducted with R, and the Bonferroni correction for multiple comparisons was applied.

### Homopolymer length variation in the plastid maturase K gene

A truncated form of the plastid maturase K (*matK*) was identified in two published (*A*. *austrokoreense* (NC_031410) and *A*. *chiisanense* (NC_029829)) plastomes from tribe Delphinieae. A primer set was designed to amplify a product around the homopolymer site for *A. austrokoreense* (matKF: GGTTCAAATCCTTCGTTGTTGGAT and matKR: ATATCAGAATCGGATGAATCGGCC). Five gDNAs of *A. austrokoreense* were isolated from herbarium specimens (Supplementary Table S7). PCRs were performed as described above using Solg *Pfu* DNA polymerase (Solgent Co., Daejeon, South Korea) to reduce the error rate at homopolymers. PCR amplification, purification, cloning and sequencing were performed as described above.

The CDD was also used to detect conserved domains. The nucleotide sequences of the plastid *matK* copies were aligned with MUSCLE^74^ in Geneious R7.

## Supplementary information

Supplementary information.
