# Supplementary material for: Recurrent gene duplication in the angiosperm tribe Delphinieae (Ranunculaceae) inferred from intracellular gene transfer events and heteroplasmic mutations in the plastid matK gene
Source: Sci Rep. 2020 Feb 17;10:2720. doi: 10.1038/s41598-020-59547-6 (PMC7026143; doi:10.1038/s41598-020-59547-6)
Supplement: Supplementary file 1 — Supplementary information. [file 41598_2020_59547_MOESM1_ESM.pdf]

Article title: Recurrent gene duplication in the angiosperm tribe Delphinieae (Ranunculaceae) inferred from intracellular gene transfer events and heteroplasmic mutations in the plastid *matK* gene

Authors: Seongjun Park, Boram An, and SeonJoo Park

Supplementary information

Figure S1. Plastome maps for tribe Delphinieae.

Figure S2. Phylogenetic relationships among 22 species of the tribe Delphinieae with two outgroups.

Figure S3. Chronogram of tribe Delphinieae divergence times.

Figure 4S. Nucleotide sequence alignments of plastid *infA*.

Figure 5S. Amino acid sequence alignment of the nuclear-encoded *rps16* copies of the four Delphinieae species with *Medicago*.

Figure 6S. Nucleotide sequence alignment of nuclear-encoded plastid *rpl32* paralogs.

Figure 7S. Inference of gene duplication events.

Figure S8. Correlation of nonsynonymous and synonymous substitution rates of the nuclear-encoded plastid *rpl32* homologs.

Figure S9. Box plots of the values of synonymous substitution rates for *Aconitum* (red) and outgroup (blue) plastid functional gene groups.

Table S1. General characteristics of newly sequenced plastomes.

Table S2. Transit peptide prediction scores of putative nuclear-encoded plastid genes with GenBank accession numbers.

Table S3. Log likelihood scores used in likelihood ratio tests (LRTs) to test the fit of model H_1 (d_N/d_S values allowed to change in a branch or within a clade) to H_0 (universal d_N/d_S values across entire tree) for *rpl32*.

Table S4. Material information

Table S5. GenBank accession numbers for taxa used in this study.

Table S6. Best partitioning scheme and evolutionary model for each partition.

Table S7. Species used for plastomes, *rpl32* and *matK* analyses.

Figure S1. Plastome maps for tribe Delphinieae. Thick lines on the inner circle indicate the inverted repeats (IR_A and IR_B), which separate the genome into small (SSC) and large (LSC) single-copy regions. Genes on the inside and outside of the map are transcribed in clockwise and counterclockwise directions, respectively. The ring of bar graphs on the inner circle indicates the GC content in dark grey. φ denotes a pseudogene and an arrow indicates the position of the *rpl32* or *rps16* genes. A) Plastome maps for four Delphinieae. The organization and gene content are identical except for the plastid-encoded *rps16* gene (a to d). B) Plastome map for *Nigella*.

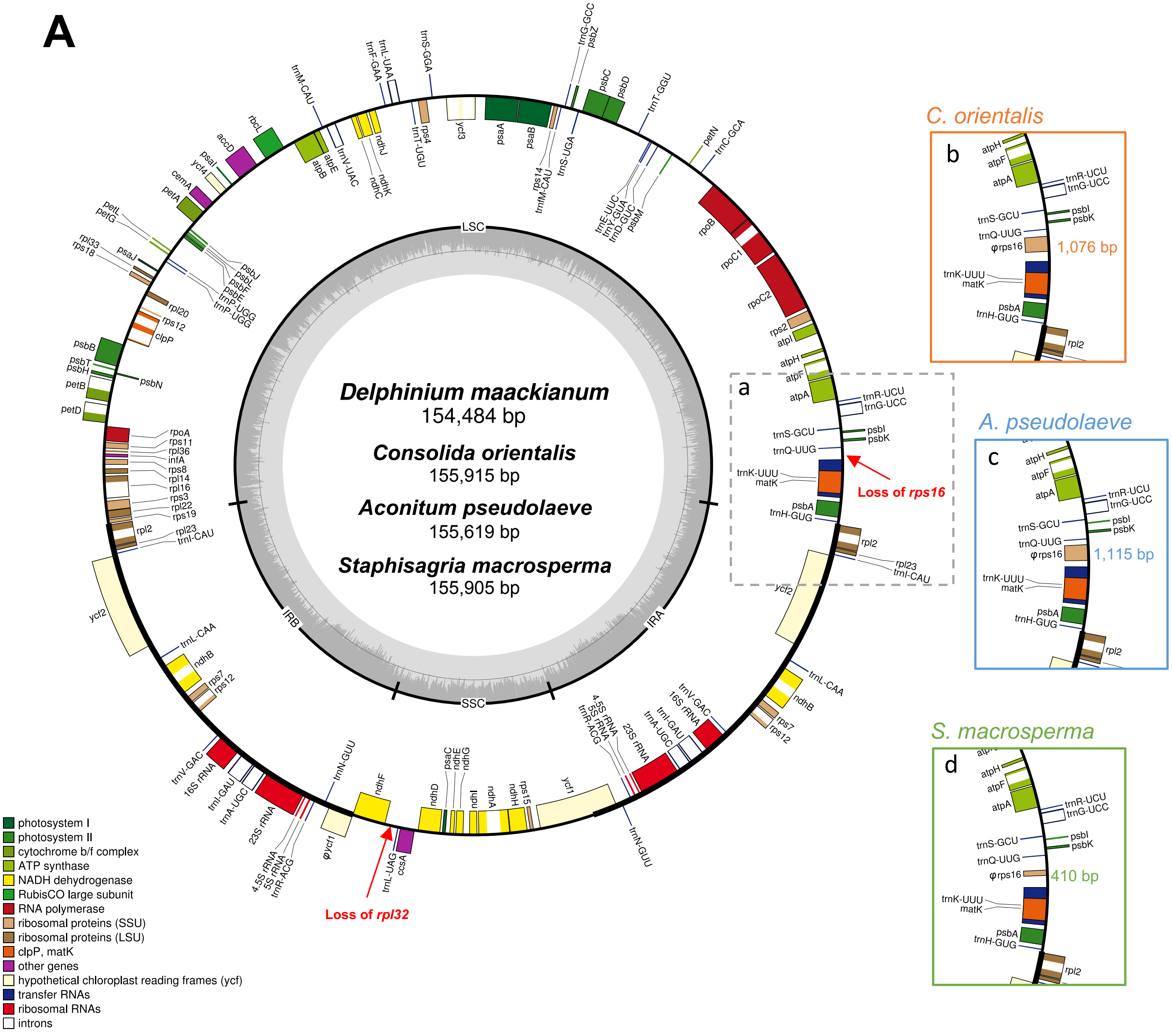

Figure S1. (continued)

B

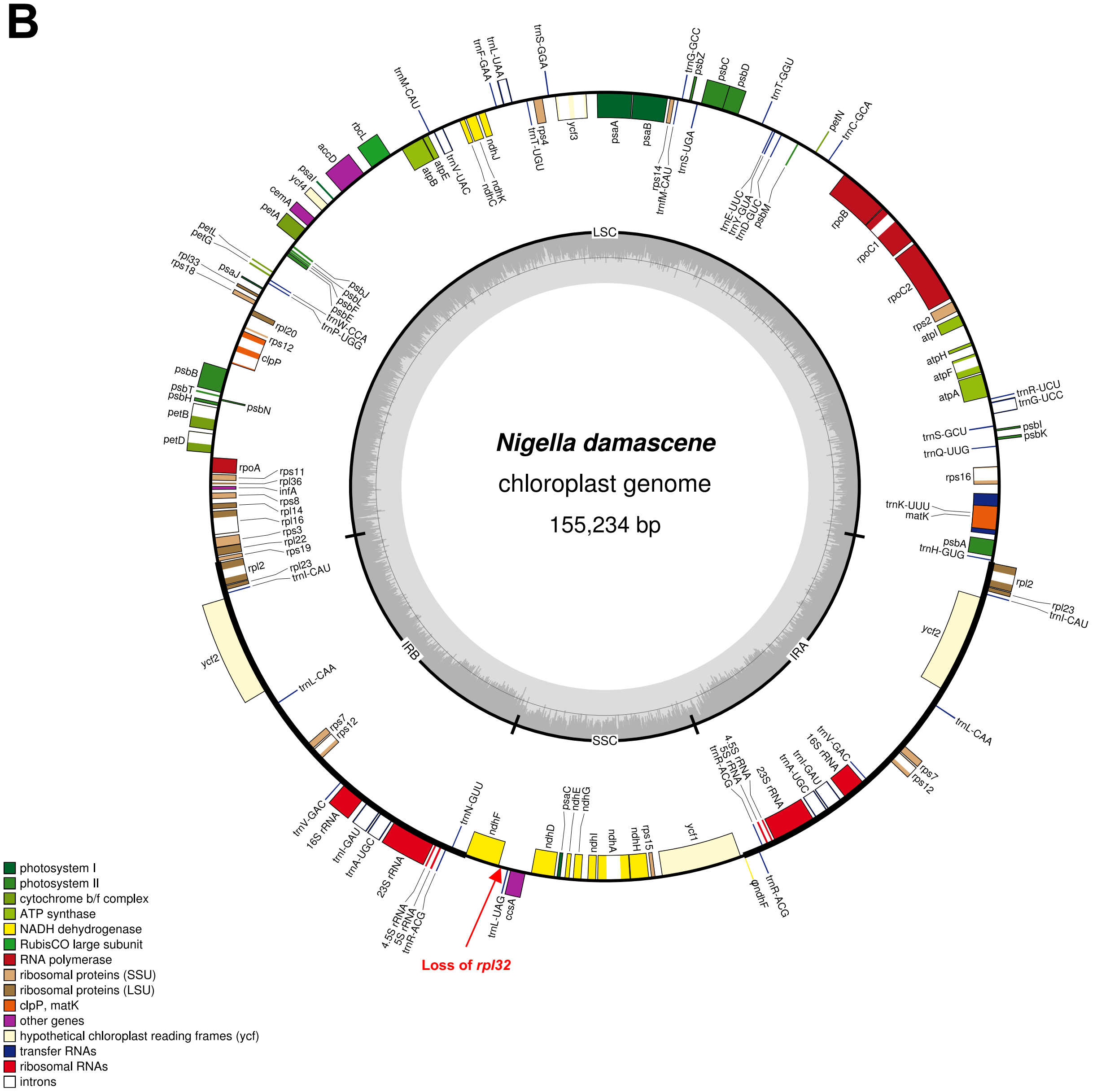

Figure S2. Phylogenetic relationships among 22 species of the tribe Delphinieae with two outgroups. The maximum likelihood tree was constructed using nucleotide sequence of 77 plastid genes. Bootstrap support values > 50% are shown at nodes.

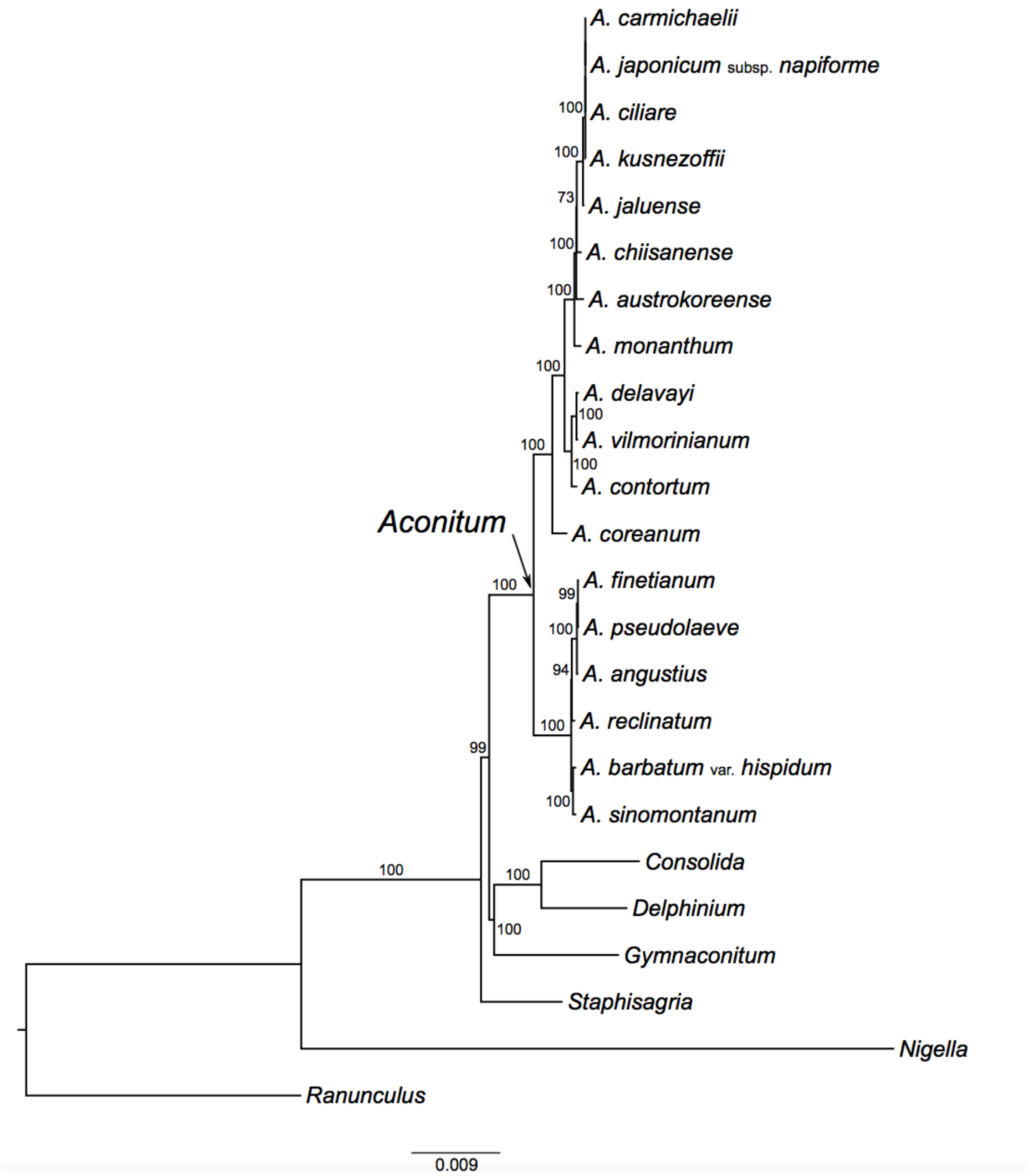

Figure S3. Chronogram of Delphinieae divergence times. Times shown are the median age estimates (Million years ago, Mya) from the BEAST analysis (blue bars indicate 95% highest posterior density [HPD] intervals).

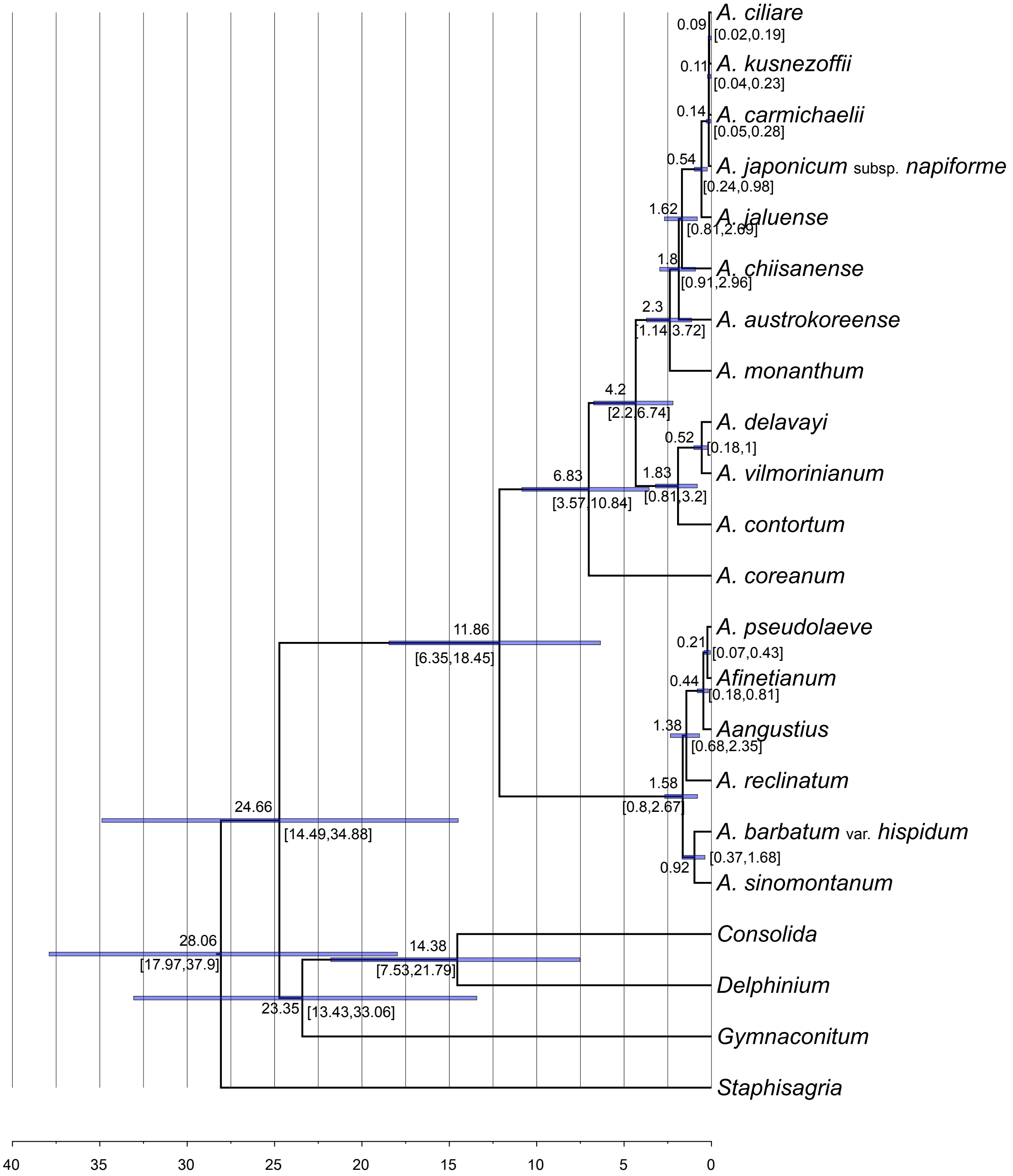

Figure S4. Nucleotide sequence alignments of plastid *infA*. Plastid *infA* sequences were extracted from 18 *Aconitum* with related species. Alignment was performed in Geneious R7. The *infA* sequences of *A. angustius*, *A. finetianum*, *A. sinomontanum*, and *Gymnaconitum gymnandrum* are annotated as gene losses, but re-analyses suggest functionality for all copies (red boxes).

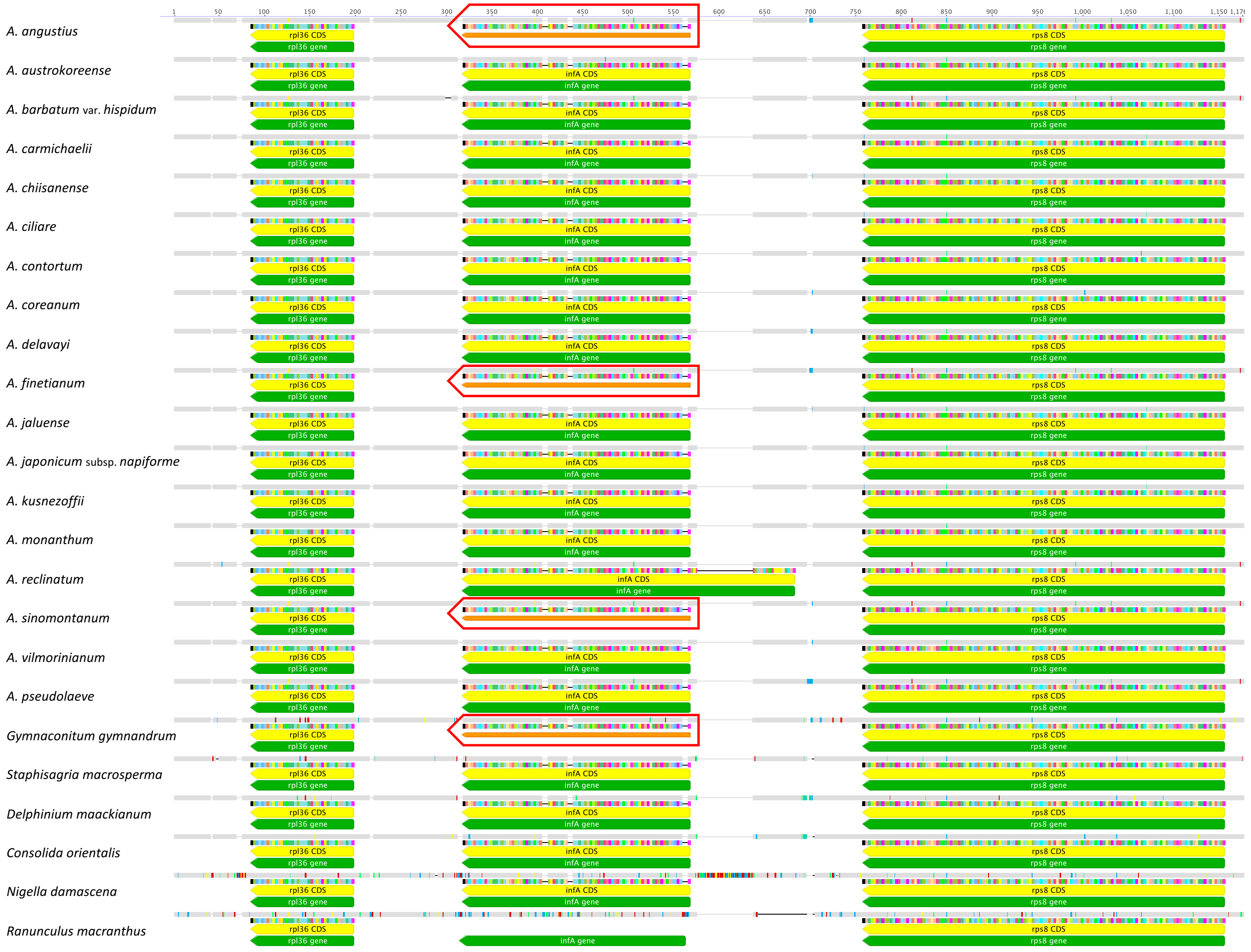

Figure S5. Amino acid sequence alignment of the nuclear-encoded *rps16* copies of the four Delphinieae species with *Medicago*. The tree was inferred using the maximum likelihood method by IQ-TREE. The numbers “1” and “2” after each species in the phylogram represent paralogs of the nuclear-encoded *rps16*. NUMT indicates a nuclear-encoded mitochondrial DNA, and NUPT indicates a nuclear-encoded plastid DNA. The sequence logo indicates the most conserved bases at each position. Red boxes indicate the conserved domain of ribosomal protein S16. Bootstrap support values are shown on the branches.

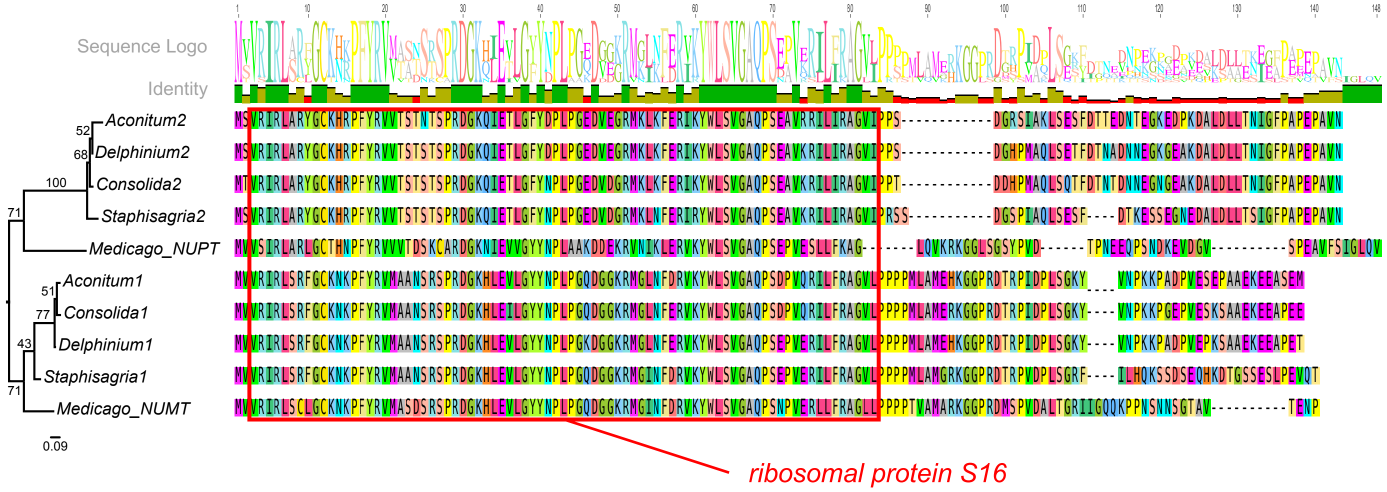

Figure S6. Nucleotide sequence alignment of nuclear-encoded plastid *rpl32* paralogs. Nucleotide and amino acid sequence alignments of the nuclear-encoded *rpl32* copies of *A. carmichaelii* (A), *A. pseudolaeve* (B), *A. ciliare* (C), *A. coreanum* (D), *A. monanthum* (E), *A. kusnexoffii* (F), *A. glandulosum* (G), *A. austrokoreense* (H), *A. japonicum* (I), and *A. barbatum* (J). The numbers in red and gray next to the alignments represents cDNA sequences and pseudogene sequences, respectively.

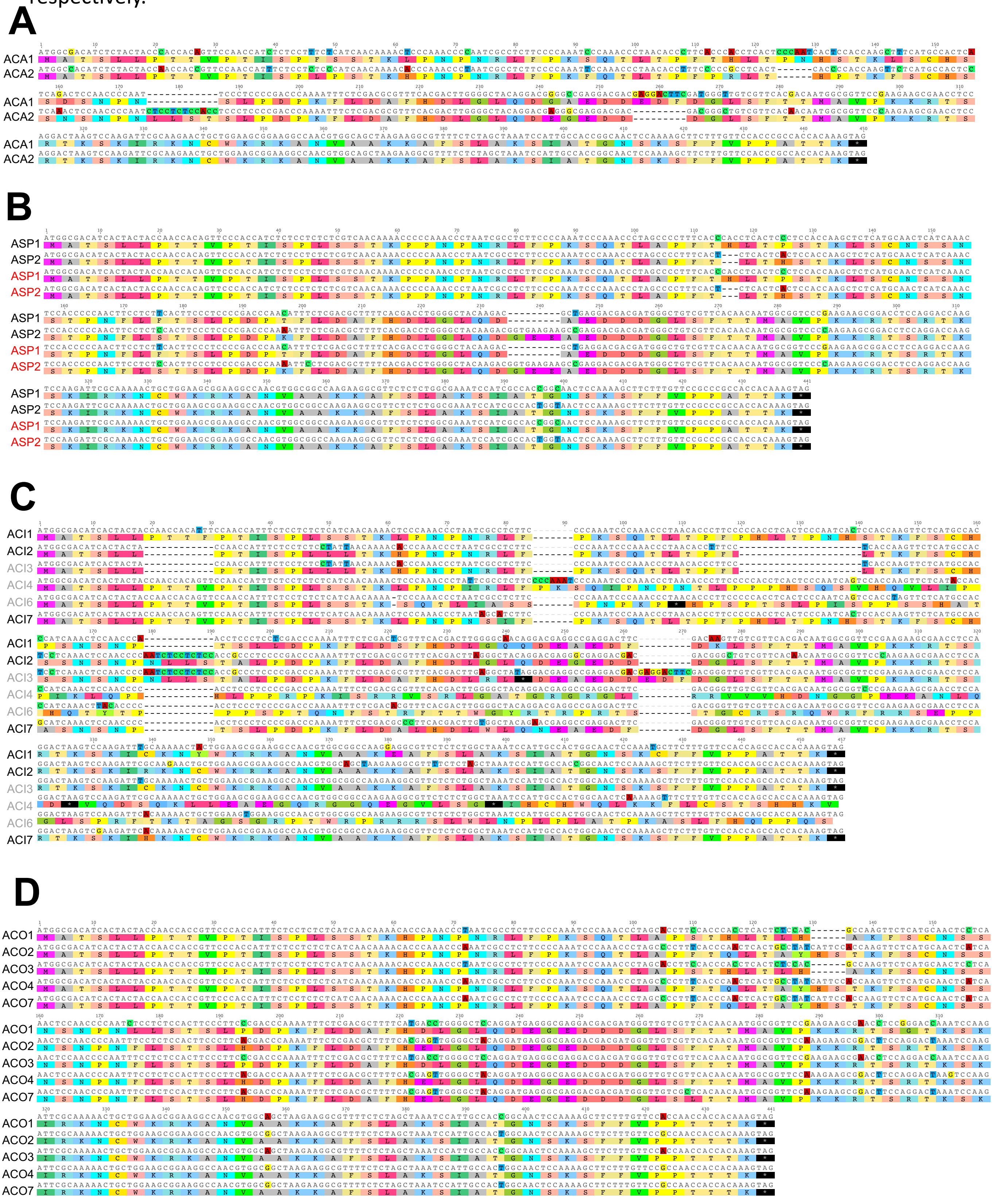

Figure S6. (continued)

E

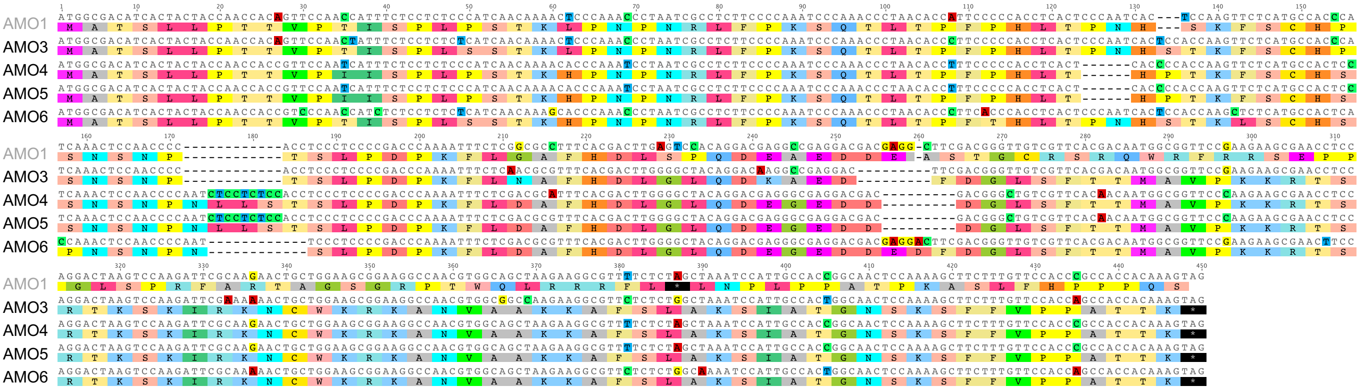

F

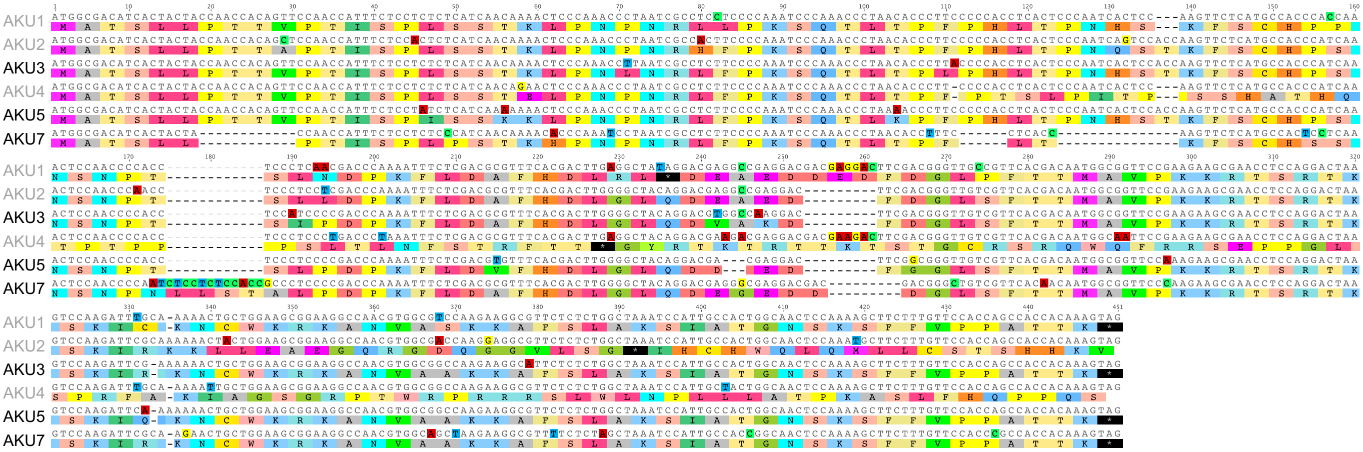

G

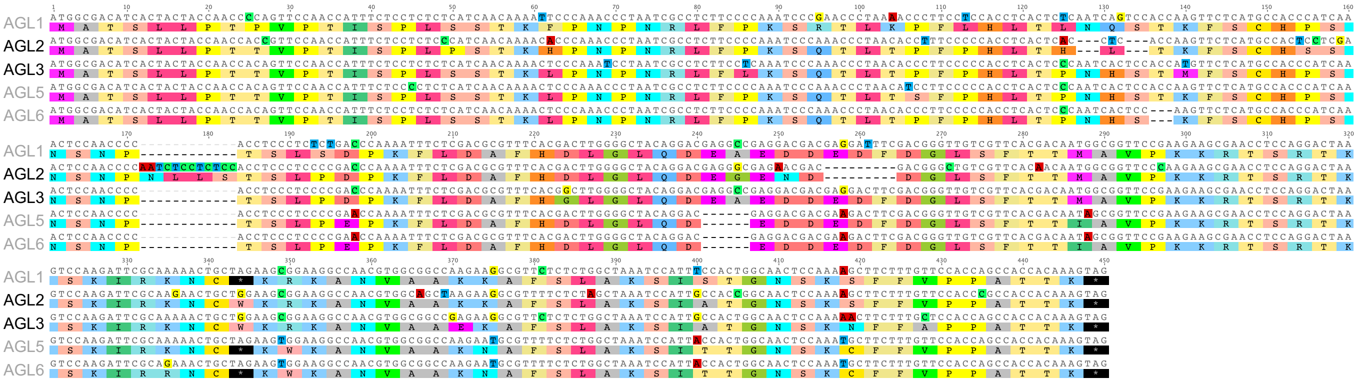

Figure S6. (continued)

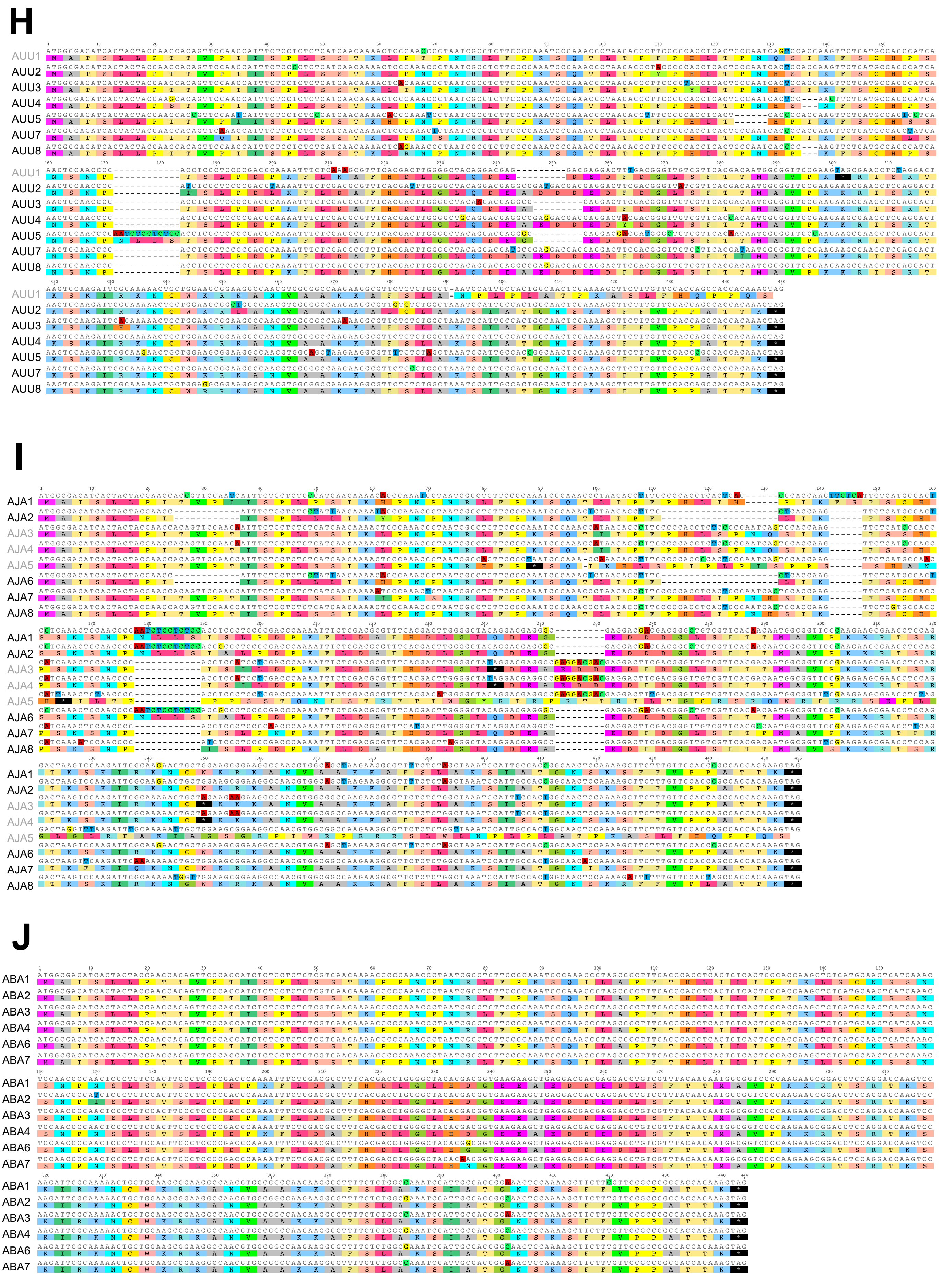

Figure S7. Inference of gene duplication events. ML gene tree based on the nuclear-encoded *rpl32* nucleotide sequences. The numbers after each species indicate paralogs of *rpl32*. Bootstrap support values > 70% are shown on the branches. Stars indicate the inferred gene duplication events. The colored lines and boxes correspond to each lineage. A) Possible topology of the duplicated homolog tree. Gray indicates gene loss or pseudogenization.

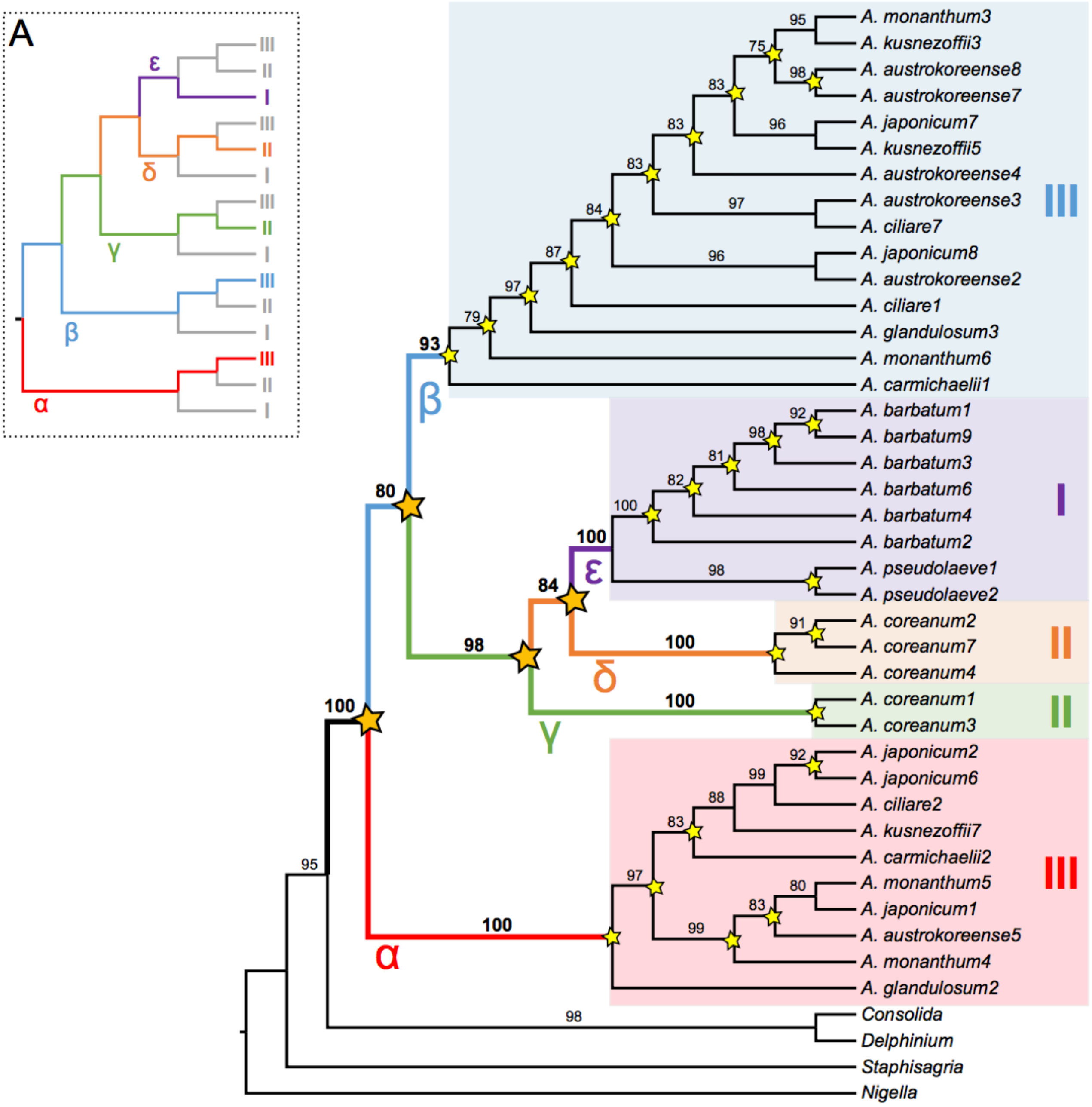

Figure S8. Correlation of nonsynonymous and synonymous substitution rates of the nuclear-encoded plastid *rpl32* homologs. The solid red line indicates the d_N/d_S ratio is equal to one. The dashed line represents the regression, which was analyzed using d_N and d_S for all homologs. The colored circles indicate branch values corresponding to five lineages.

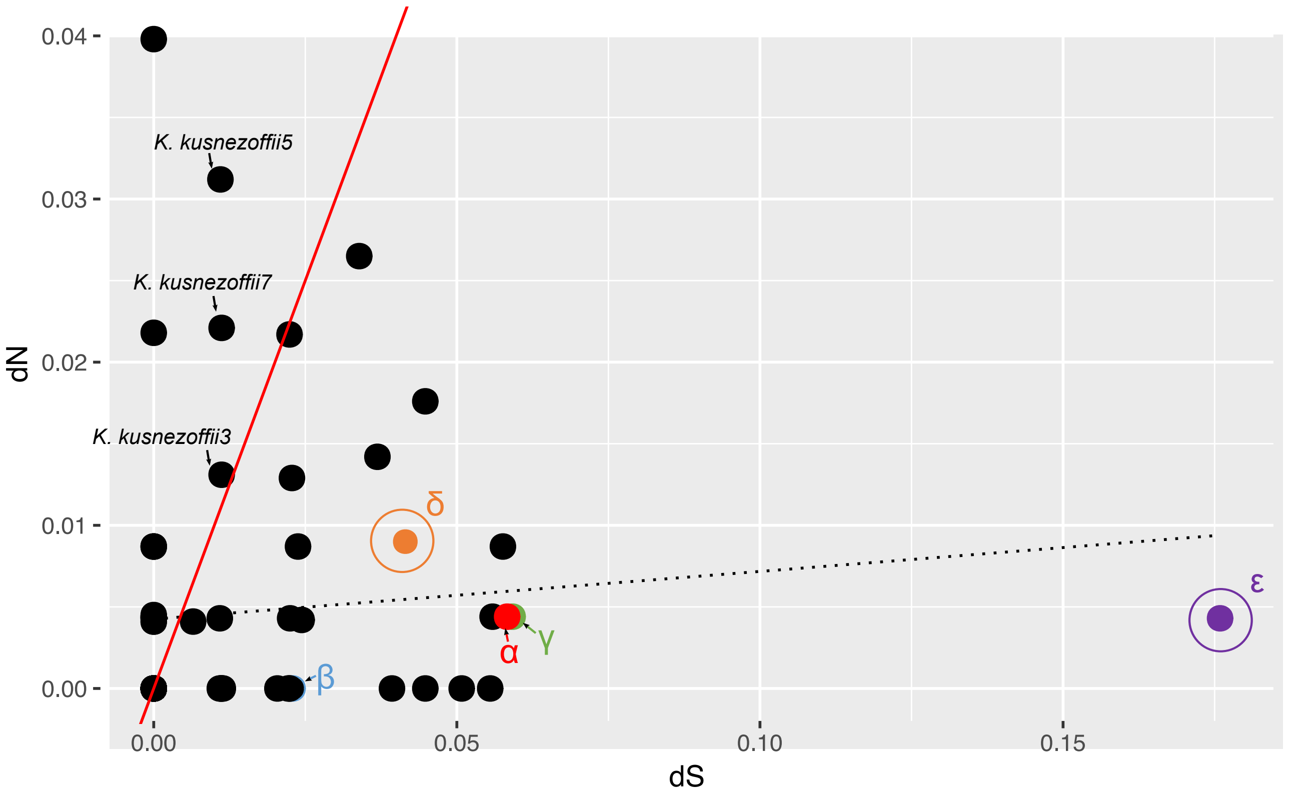

Figure S9. Box plots of the values of synonymous substitution rates for *Aconitum* (red) and outgroup (blue) plastid functional genes groups . The box represents values between quartiles, the solid lines extend to the minimum and maximum values, outliers are shown as circles and horizontal lines in the boxes show the median values. Significance of fit was evaluated by Wilcoxon rank sum tests in the R package.

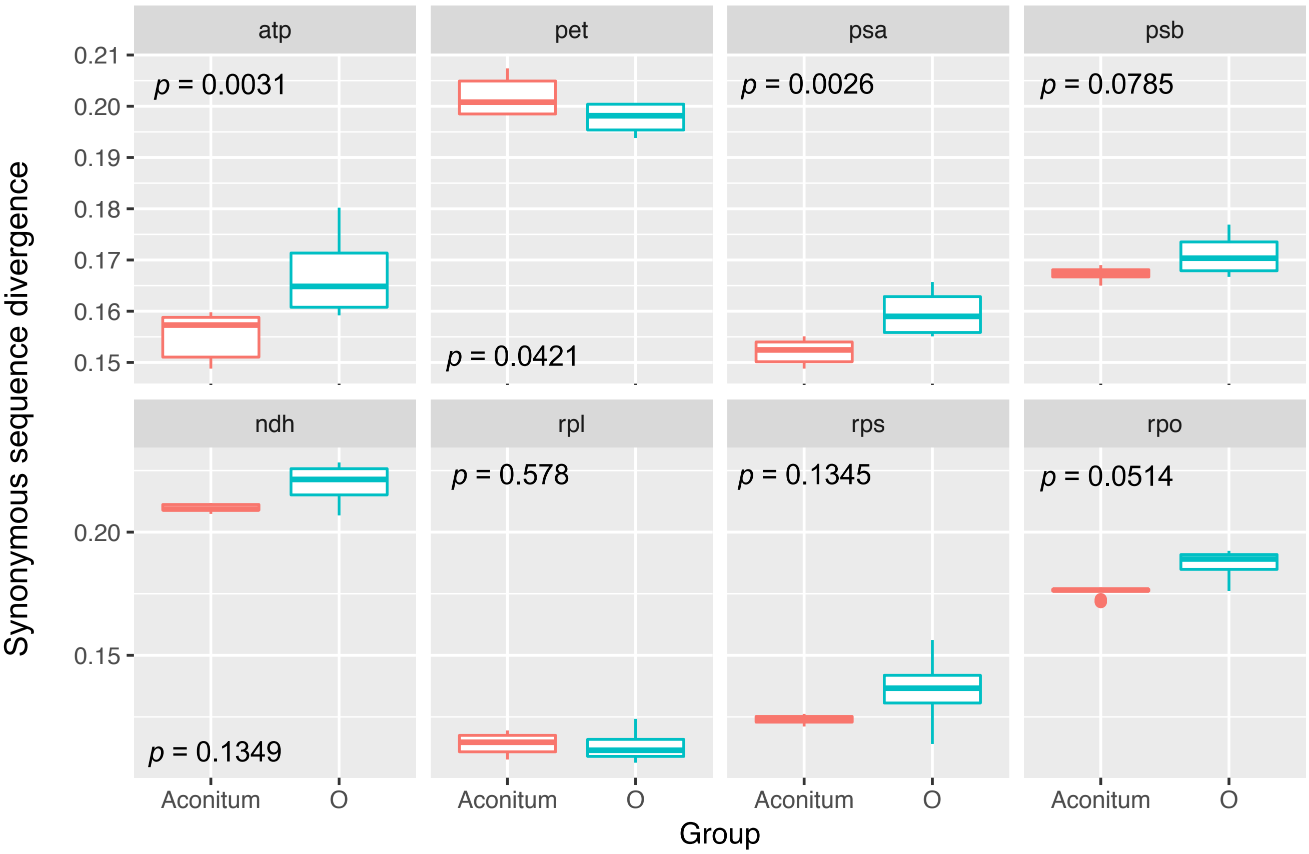

Table S1. General characteristics of newly sequenced plastomes.

	Aconitum pseudolaeve	*Consolida orientalis*	*Delphinium maackianum*	*Staphisagria macrosperma*	*Nigella damascene*
Genome size (bp)	155,619	155,915	154,484	155,905	155,234
LSC size (bp)	86,679	85,915	85,055	86,577	87,119
IR size (bp)	25,927	26,786	26,564	26,100	25,167
SSC size (bp)	17,086	16,428	16,301	17,128	17,781
GC content (%)	38.0	38.3	38.1	38.1	38.8
Protein genes	77	77	77	77	78
rRNAs	4	4	4	4	4
tRNAs	30	30	30	30	30
Coverage	515x	1,004x	831x	837x	372x
Genbank accession number	MN648400	MN648401	MN648402	MN648404	MN648403

Table S2. Transit peptide prediction scores of putative nuclear-encoded plastid genes with GenBank accession numbers. cTP = chloroplast transit peptide. mTP = a mitochondrial targeting peptide. RC indicates reliability class, from 1 to 5, where 1 indicates the strongest prediction. Tplen means predicted presequence length (cleavage sites). Bold font indicates prediction of localization (chloroplast or mitochondrion).

Gene	Taxa	Len	cTP	mTP	SP	other	Loc	RC	TPlen	GenBank
nuclear	*A. austrokoreense2*	145	**0.923**	0.083	0.022	0.121	C	1	30	MN652017
encoded	*A. austrokoreense3*	142	**0.917**	0.113	0.019	0.100	C	1	43	MN652018
rpl32	*A. austrokoreense4*	144	**0.963**	0.052	0.025	0.069	C	1	52	MN652019
for plastids	*A. austrokoreense5*	144	**0.970**	0.048	0.017	0.081	C	1	30	MN652020
	A. austrokoreense7	145	**0.943**	0.111	0.019	0.065	C	1	49	MN652021
	A. austrokoreense8	144	**0.946**	0.100	0.020	0.060	C	1	30	MN652022
	A. barbatum1	147	**0.970**	0.044	0.015	0.086	C	1	48	MN652023
	A. barbatum2	147	**0.966**	0.048	0.016	0.089	C	1	48	MN652024
	A. barbatum3	147	**0.970**	0.044	0.015	0.086	C	1	48	MN652025
	A. barbatum4	147	**0.970**	0.044	0.015	0.086	C	1	48	MN652026
	A. barbatum6	147	**0.971**	0.045	0.014	0.082	C	1	48	MN652027
	A. barbatum9	147	**0.971**	0.046	0.014	0.081	C	1	48	MN652028
	A. carmichaelii1	145	**0.959**	0.081	0.025	0.082	C	1	49	SRR6225422
	A. carmichaelii2	144	**0.967**	0.070	0.011	0.074	C	1	39	SRR6225422
	A. ciliare1	142	**0.948**	0.073	0.019	0.080	C	1	43	MN652029
	A. ciliare2	135	**0.809**	0.153	0.035	0.093	C	2	26	MN652030
	A. ciliare7	142	**0.948**	0.062	0.013	0.109	C	1	51	MN652031

A. coreanum1	144	**0.975**	0.052	0.013	0.069	C	1	47	MN652032
A. coreanum2	146	**0.932**	0.074	0.019	0.110	C	1	49	MN652033
A. coreanum3	144	**0.975**	0.053	0.014	0.071	C	1	47	MN652034
A. coreanum4	146	**0.932**	0.074	0.019	0.110	C	1	49	MN652035
A. coreanum7	146	**0.931**	0.074	0.018	0.111	C	1	49	MN652036
A. japonicum1	146	**0.966**	0.045	0.016	0.077	C	1	49	MN652037
A. japonicum2	135	**0.695**	0.179	0.041	0.098	C	3	26	MN652038
A. japonicum6	135	**0.809**	0.153	0.035	0.093	C	2	26	MN652039
A. japonicum7	142	**0.959**	0.061	0.015	0.080	C	1	43	MN652040
A. japonicum8	142	**0.936**	0.086	0.018	0.094	C	1	43	MN652041
A. kusnezoffii3	142	**0.921**	0.107	0.015	0.094	C	1	30	MN652042
A. kusnezoffii5	141	**0.971**	0.044	0.017	0.129	C	1	43	MN652043
A. kusnezoffii7	135	**0.893**	0.105	0.029	0.127	C	2	26	MN652044
A. monanthum3	142	**0.947**	0.073	0.013	0.089	C	1	43	MN652045
A. monanthum4	144	**0.970**	0.048	0.017	0.081	C	1	30	MN652046
A. monanthum5	144	**0.970**	0.048	0.017	0.081	C	1	30	MN652047
A. monanthum6	145	**0.960**	0.079	0.020	0.079	C	1	49	MN652048
A. pseudolaeve1	143	**0.972**	0.039	0.014	0.084	C	1	47	MN652049
A. pseudolaeve2	145	**0.975**	0.056	0.015	0.078	C	1	46	MN652050
A. glandulosum2	144	**0.963**	0.060	0.013	0.086	C	1	30	MN652051
A. glandulosum3	145	**0.942**	0.081	0.023	0.086	C	1	30	MN652052
Consolida orientalis	149	**0.965**	0.072	0.010	0.038	C	1	49	MN652053
Delphinium maackianum	145	**0.953**	0.054	0.040	0.053	C	1	48	MN652054
Staphisagria macrosperma	152	**0.936**	0.157	0.018	0.030	C	2	45	MN652055

	Nigella damascena	165	**0.866**	0.305	0.015	0.032	C	3	25	SRR341997
nuclear	*A. carmichaelii*	131	0.018	**0.886**	0.010	0.254	M	2	7	SRR6225422
encoded	*Consolida orientalis*	131	0.024	**0.926**	0.008	0.171	M	2	7	MN652011
rps16	*Delphinium maackianum*	131	0.024	**0.910**	0.009	0.171	M	2	7	MN652013
for plastids	*Staphisagria macrosperma*	128	0.027	**0.907**	0.006	0.196	M	2	7	MN652015
nuclear	*A. carmichaelii*	134	0.023	**0.853**	0.015	0.189	M	2	7	SRR6225422
encoded	*Consolida orientalis*	134	0.020	**0.861**	0.012	0.196	M	2	7	MN652012
rps16	*Delphinium maackianum*	134	0.024	**0.858**	0.013	0.180	M	2	7	MN652014
for mitochondria	*Staphisagria macrosperma*	136	0.024	**0.822**	0.011	0.201	M	2	7	MN652016

Table S3. Log likelihood scores used in likelihood ratio tests (LRTs) to test the fit of model H_1 (d_N/d_S values allowed to change in a branch or within a clade) to H_0 (universal d_N/d_S values across entire tree) for *rpl32*. (H_0 = -2322.2505)

Taxa	dN/dS	inL H1	2*(H1-H0)	p-value	d.f.	Bonferroni
A. kusnezoffii3	1.1707	-2320.957	2.586998	0.1077444	1	1.0000
A. kusnezoffii5	2.8352	-2317.3939	9.713194	0.0018295	1	0.1482
A. japonicum7	1.9710	-2319.2535	5.993944	0.0143551	1	1.0000

Table S4. Material information

Taxon	Voucher Source/Hebraium
Aconitum pseudolaeve	Park S 201645/YNUH
Consolida orientalis	Park S 201711/YNUH
Delphinium macckianum	Park S 201646/YNUH
Staphisagria macrosperma	Park S 201712/YNUH
Nigella damascena	Park S 201713/YNUH

Table S5. GenBank accession numbers for taxa used in this study.

Taxa		Genbank accession number
Aconitum	*angustius*	NC_036357
Aconitum	*austrokoreense*	NC_031410
Aconitum	*barbatum* var. *hispidum*	KT820664
Aconitum	*carmichaelii*	NC_030761
Aconitum	*chiisanense*	NC_029829
Aconitum	*ciliare*	NC_031420
Aconitum	*contortum*	MG678803
Aconitum	*coreanum*	NC_031421
Aconitum	*delavayi*	MG678802
Aconitum	*finetianum*	NC_036358
Aconitum	*jaluense*	KT820669
Aconitum	*japonicum* subsp. *napiforme*	KT820670
Aconitum	*kusnezoffii*	NC_031422
Aconitum	*monanthum*	NC_031423
Aconitum	*reclinatum*	MF186593
Aconitum	*sinomontanum*	NC_036359
Aconitum	*vilmorinianum*	MG678799
Aconitum	*pseudolaeve*	MN648400
Staphisagria	*macrosperma*	MN648404
Delphinium	*maackianum*	MN648402
Consolida	*orientalis*	MN648401
Gymnaconitum	*gymnandrum*	NC_033341
Nigella	*damascena*	MN648403
Ranunculus	*macranthus*	NC_008796

Table S6. Best partitioning scheme and evolutionary model for each partition.

No.	Gene or gene groups	Best-fit model	Best-fit partitioning scheme
1	*accD*	TPM3u+F	TPM3u+F
2	*atp*	K3Pu+F+G4	GTR+F+R3
3	*ccsA*	TPM3u+F+G4	TVM+F+G4
4	*cemA*	HKY+F	TVM+F+G4
5	*clpP*	TIM3e	TPM3u+F
6	*ndh*	TVM+F+G4	TVM+F+G4
7	*pet*	TPM3+F+I	GTR+F+I
8	*psa*	K3Pu+F+I	GTR+F+I
9	*psb*	TIM+F+I	GTR+F+I
10	*rbcL*	K3P+I	GTR+F+I
11	*rpl*	TPM3u+F+G4	GTR+F+R3
12	*rpo*	GTR+F+G4	GTR+F+R3
13	*rps*	TIM+F+G4	GTR+F+R3
14	*ycf1*	TVM+F+R2	TVM+F+R2
15	*ycf2*	TVM+F+G4	TVM+F+G4
16	*ycf3*	K3Pu+F	TVM+F+G4
17	*ycf4*	K3Pu+F	GTR+F+I

Table S7. Species used for plastomes, *rpl32* and *matK* analyses

Taxa	Geographic regions	Sample source
A. austrokoreense	Korea	KH/KEB1150777, KEB1434286, KEB1165044, KEB1051829, KEB1164289
A. barbatum	Mongolia	YNUH/1404
A. ciliare	Korea	KH/KHB1345877
A. coreanum	Korea	KH/KHB1345877
A. glandulosum	Mongolia	YNUH/1432
A. japonicum	Korea	YNUH/sj1707
A. kusnezoffii	Korea	YNUH/sj1713
A. monanthum	Korea	KH/KHE1272784
A. pseudolaeve	Korea	YNUH/sj1704
